# Organic LEDs Based on Bis(8-hydroxyquinoline) Zinc Derivatives with a Styryl Group

**DOI:** 10.3390/molecules28217435

**Published:** 2023-11-05

**Authors:** Malgorzata Sypniewska, Monika Pokladko-Kowar, Ewa Gondek, Aleksandra Apostoluk, Piotr Kamedulski, Vitaliy Smokal, Peng Song, Junyan Liu, Robert Szczesny, Beata Derkowska-Zielinska

**Affiliations:** 1 Institute of Physics, Faculty of Physics, Astronomy and Informatics, Nicolaus Copernicus University in Torun, Grudziadzka 5, 87-100 Torun, Poland; msyp@doktorant.umk.pl; 2 Department of Physics, Cracow University of Technology, Podchorążych Str. 1, 30-084 Krakow, Poland; mpokladkokowar@pk.edu.pl (M.P.-K.); egondek@pk.edu.pl (E.G.); 3 Institut des Nanotechnologies de Lyon (INL-UMR5270), Université de Lyon, INSA-Lyon, ECL, UCBL, CPE, CNRS, 69621 Villeurbanne, France; aleksandra.apostoluk@insa-lyon.fr; 4 Faculty of Chemistry, Nicolaus Copernicus University in Torun, Gagarina 7, 87-100 Torun, Poland; pkamedulski@umk.pl (P.K.); roszcz@umk.pl (R.S.); 5 Centre for Modern Interdisciplinary Technologies, Nicolaus Copernicus University, Wilenska 4, 87-100 Torun, Poland; 6 Department of Chemistry, Taras Shevchenko National University of Kyiv, 60 Volodymyrska, 01033 Kyiv, Ukraine; vitaliismokal@gmail.com; 7 State Key Laboratory of Robotics and System, Harbin Institute of Technology, Harbin 150001, China; songpeng@hit.edu.cn (P.S.); ljywlj@hit.edu.cn (J.L.); 8 School of Instrumentation Science and Engineering, Harbin Institute of Technology, Harbin 150001, China; 9 School of Mechatronics Engineering, Harbin Institute of Technology, Harbin 150001, China

**Keywords:** OLED, thin films, bis(8-hydroxyquinoline) zinc derivatives, ellipsometry, absorption coefficient, refractive index, photo- and electroluminescence

## Abstract

For the first time, organic light-emitting diodes (OLEDs) based on bis(8-hydroxyquinoline) zinc with a styryl group (ZnStq) dispersed in poly(N-vinylcarbazole) matrix (ZnStq_R:PVK, where R = H, Cl, OCH_3_) were fabricated. The ZnStq_R:PVK films made via the spin-coating method were used as the active layer in these devices. The produced OLEDs showed strong electroluminescence with yellow emissions at 590, 587 and 578 nm for the ZnStq_H:PVK, ZnStq_Cl:PVK and ZnStq_OCH_3_:PVK, respectively. For all the studied thin films, the main photoluminescence emission bands were observed between 565 and 571 nm. The OLED with the ZnStq_OCH_3_:PVK layer with a narrow electroluminescence spectrum was found to have sufficient color purity to produce ultra-high-resolution displays with reduced power consumption (full width at half maximum of 59 nm, maximum brightness of 2244 cd/m^2^ and maximum current efficiency of 1.24 cd/A, with a turn-on voltage of 6.94 V and a threshold voltage of 7.35 V). To characterize the photophysical properties of the active layer, the ZnStq_R:PVK layers samples were additionally deposited on glass and silicon substrates. We found that the obtained results predestine ZnStq_R:PVK layers for use in the lighting industry in the future.

## 1. Introduction

Organic light-emitting diodes (OLEDs) have attracted the attention of researchers for many years, resulting in improved optoelectronic properties, such as the brightness and operating voltage. This development has also led to the widespread use of these diodes. However, there are still many things to improve, such as the lifetime and stability of these devices.

The 8-hydroxyquinoline derivatives are a group of compounds with rich and varied activity. These compounds incorporate the 8-hydroxyquinoline moiety, which is a bicyclic fragment consisting of a pyridine and phenol ring [1]. 8-hydroxyquinoline and its derivatives, especially Alq_3_ (tris(8-hydroxyquinoline)aluminum) and Znq_2_ (bis(8-hydroxyquinoline) zinc), have gained wider interest, as they may find applications in various domains, such as chromatography, electrochemiluminescence, photodiodes, optical waveguides, lasers, photoswitches, solar cells and organic light-emitting diodes [2,3,4,5,6,7]. Metal 8-hydroxyquinoline (Mq_n_, i.e., Liq, Caq_2_, Mnq_2_, Znq_2_, Alq_3_, Biq_3_ and Irq_3_) complexes (in which n is the oxidation state of the metal (M)) are widely studied for their excellent luminescent, field emission, charge transport, photoconductivity, and photo-switching properties [8,9], which depend, in particular, on the nature of the metal ion, aggregation, molecular and crystal structures and intermolecular non-covalent interactions [10]. Among Mq_n_, bis(8-hydroxyquinoline) zinc (Znq_2_) is a good candidate to improve the luminescent properties of OLEDs [11,12,13,14] due to the fact that it has a high electroluminescence quantum yield, good color tunability properties, and good thermal stability for low-operating-voltage OLEDs [15].

Styrylquinolines (Stqs) and their derivatives belong to the class of stilbenes and can undergo various photochemical reactions [16,17]. Importantly, they have two active fragments: an ethylene group and an endocyclic nitrogen atom. Formerly, styrylquinoline dyes were used for various sensitive materials, such as sensitizers or desensitizers [17]. The development of new technologies has allowed for the discovery of new applications of styrylquinoline dyes for electroluminescence [18] and photochromism [19,20]. The photochemical properties of 2-styrylquinoline and its derivatives indicate that the substituents in the styryl group increase the photoisomerization quantum yield [21]. In our previous paper on the luminescence and structural properties of styrylquinoline copolymers with different substituents [22], we showed that all the investigated styrylquinolines are promising materials for applications in organic light-emitting diodes.

Currently, Znq_2_ derivatives are becoming more and more popular among scientists who use them in organic light-emitting diodes. Sharma et al. presented the chlorine-substituted zinc quinolate Zn(Cl_2_q)_2_, which, in the structure ITO/TPD/Zn(Cl_2_q)_2_/Alq_3_/LiF/Al, showed maximum electroluminescence at 545 nm, and its maximum brightness was 363 cd/m^2^ [23]. Ouyang et al. synthesized zinc complexes of 8-hydroxyquinoline with a 2-substituted carbazole group in position 2 (E)-2-(2-(9-p-tolyl-9H-carbazol-3-yl)vinyl)quinolatozine, obtaining a brightness of 3596 cd/m^2^ at a maximum current efficiency equal to 1.76 cd/A [24]. The above-mentioned complexes showed multifunctional light-emitting and hole-transporting properties. Virender et al. synthesized a mixed complex of Zn(Bpy)q (zinc (2,2′-bipyridine) 8-hydroxyquinoline) and, for the structure ITO/α-NPD/Zn(Bpy)q/Alq_3_/LiF/Al, electroluminescence was obtained at 545 nm, and the brightness of the diode was 1030 cd/m^2^ at the maximum current efficiency (1.34 cd/A) [25]. Carbazole-substituted 8-hydroxyquinoline zinc complexes have been described by He Ping Zeng et al. [26] with substituted 8-hydroxyquinoline (electron transport) and carbazole (hole transport) ligands due to the facilitated recombination of charge carriers, which contributes to the improvement in the electroluminescent properties. The luminance of the device produced using the complexes was 402 and 489 cd/m^2^. Kapoor et al. synthesized complexes of 2-(2-pyridyl)benzimidazole (PBI) with a 5,7-dimethyl, 8-hydroxyquinoline ligand [(Me_2_q)_2_Zn] and (Me_2_q)(PBI)Zn] [27], which produced yellow photoluminescence at 571 and 560 nm. Through research, the light-emitting properties of zinc complexes have been further improved. The produced device in the ITO/α-NPD/zinc complex/BCP/Alq_3_/LiF/Al configuration gives electroluminescence at 572 and 562 nm. Nishal et al. [28] fabricated ITO/α-NPD/zinc complex/BCP/Alq_3_/LiF/Al devices using the BTZ (2-(2-hydroxyphenyl)benzothiazole) ligand with various zinc 8-hydroxyquinoline derivatives, which emit light with wavelengths of 532, 572 and 541 nm. The purpose of using these complexes was to fine-tune the color of the emitted light using different ligands to achieve full-color displays. However, almost all of the above organic light-emitting diodes had low maximum brightness values.

The aim of this work was to make simple OLED structures (ITO/PEDOT:PSS/ZnStq_R:PVK/Al) based on newly synthesized bis(8-hydroxyquinoline) zinc with a styryl group (ZnStq_R, where R = H, Cl, OCH_3_) in poly(9-vinylcarbazole) (PVK) matrix with a higher maximum brightness value than those obtained so far for Znq_2_ derivatives. The electroluminescent properties of the obtained OLEDs were then measured. Thin films of the ZnStq_R:PVK were prepared on glass and silicon substrates using the spin-coating method. The optical and structural properties of these thin films were investigated using IR, UV-Vis, spectroscopic ellipsometry (SE) and photoluminescence (PL). Thin-film measurements were important in this study because they allowed us to measure the spectroscopic and optical properties of the newly synthesized bis(8-hydroxyquinoline) zinc with a styryl fragment independently of the rest of the diode structure. 

## 2. Results and Discussion

**Figure 1:**
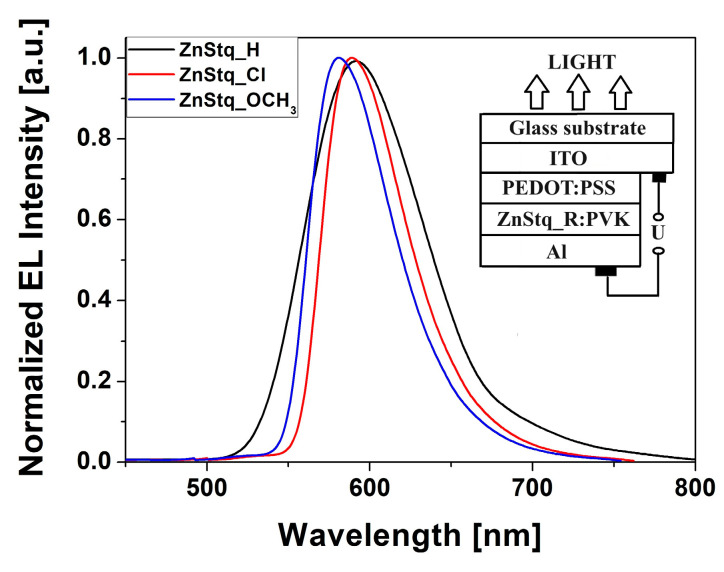
Electroluminescence spectra of ITO/PEDOT:PSS/ZnStq_R:PVK/Al structures. Inset: studied organic light-emitting diode structure.

The electroluminescence (EL) spectra of the prepared, simple organic light-emitting diodes (OLEDs) are shown in Figure 1. It can be seen that the organic light-emitting diodes exhibited strong yellow electroluminescent emissions with peaks at 590 nm, 587 nm and 578 nm for the ZnStq_H:PVK, ZnStq_Cl:PVK and ZnStq_OCH_3_:PVK, respectively (see also Table 1). We found that the presence of the electron-acceptor Cl group in the structure of the ZnStq causes a slight shift of the EL maximum towards shorter wavelengths. In contrast, the presence of the electron-donor group OCH_3_ causes a greater blue shift of the EL maximum. In addition, we found that organic light-emitting diodes with ZnStq layers and electron-donating or -withdrawing substituents have the narrowest electroluminescence spectra (see Table 1). The EL spectrum is the widest for the ZnStq_H sample. It is well known that the narrower the full width at half maximum (*FWHM*), the more accurate the color of the organic light-emitting diode. We assume that such a blue shift and narrowing of the EL spectra may have been caused by a decrease in the thickness of the studied layer [29]. However, it can be seen from Figure 1 that various substitutions in the ZnStq had little effect on the EL spectral features. These results indicate that the incorporation of either an electron-withdrawing or electron-donating substituent on the ZnStq has only a subtle tuning effect on the energy bandgap so that the EL of these materials is still located in the same range as that of the ZnStq_H [30]. 

The inset of Figure 1 shows the fabricated multilayer device with the following structure: glass/ITO/PEDOT:PSS/ZnStq_R:PVK/Al. The organic light-emitting diode fabrication procedure is described in Section 3.2.

**Figure 2:**
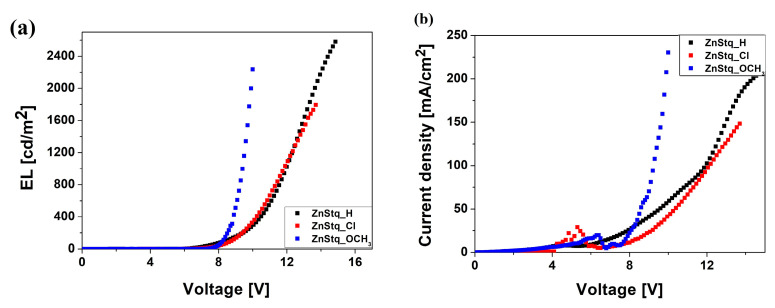
Luminance–voltage characteristics of ITO/PEDOT:PSS/ZnStq_R:PVK/Al structure (**a**); current density–voltage curves for the ITO/PEDOT:PSS/ZnStq_R:PVK/Al structure (**b**).

Figure 2a,b present the luminance–voltage and current density–voltage curves recorded for the organic EL devices prepared based on the ZnStq_R:PVK, respectively. The current density–voltage curves show the relationship between the current and voltage from the no-load point to the maximum voltage in the OLEDs. Thus, they show the efficiency of the diodes. The luminance–voltage curves show the relationship between the luminescence intensity and the voltage from the point without luminance to the point with maximum luminosity for a given voltage in the organic light-emitting diodes. Figure 2a presents the luminance–voltage characteristics, which exhibit the features typical for electroluminescent devices. For the studied organic light-emitting diodes, the turn-on voltages (*U_on_*), at which EL becomes detectable [31], are 5.38, 5.67 and 6.94 V for the ZnStq_H, ZnStq_Cl and ZnStq_OCH_3_, respectively. The highest value of the *U_on_* was observed for an electron-donating methoxy group. The values of the threshold voltage (*U_T_*), at which the device starts to switch on [32], were specified at 6.35, 7.80 and 7.35 V for the ZnStq_H, ZnStq_Cl and ZnStq_OCH_3_, respectively (see Figure 2b and Table 1). Lim et al. [33] obtained *U_on_* values between 6 and 7 V and about 7 V for the *U_T_* value for their diode ITO/TPD:PMDA-ODA PI/Znq_2_/Al, which are close to the values obtained by us for the ZnStq_R-based OLEDs.

In addition, Table 1 shows the maximum brightness (*B_max_*) values for the studied organic light-emitting diodes. We can see that the highest value was 2595 cd/m^2^ for the ZnStq_H. A similar value of the *B_max_* (= 2244 cd/m^2^) was obtained for the ZnStq with an electron-donating substituent, whereas, for the electron-withdrawing one, the maximum brightness was only 1793 cd/m^2^. Additionally, the highest values of the maximum current efficiency (*Max CE*) were determined for the neutral (ZnStq_H) and electron-donating (ZnStq_OCH_3_) substituent. For the ZnStq_Cl, the *Max CE* value is about 70% of the other values obtained. It should be mentioned that we used a simpler and cheaper method of producing the emissive layer (spin-coating) compared to the vacuum deposition methods presented in Refs. [33,34]. Rawat et al. described the OLED structure ITO/PEDOT:PSS/NPB/Znq_2_/BCP/LiF/Al, in which the emissive layer was deposited in a high vacuum (~1–5 × 10^−6^ mbar) [34]. The diode that they obtained had a maximum electroluminescence at 540 nm with a maximum current efficiency of 0.64 cd/A and a maximum brightness of 791 cd/m^2^. The *B_max_* value for the active layer with pure Znq_2_ is about 30–40% lower than the values we obtained.

**Figure 3:**
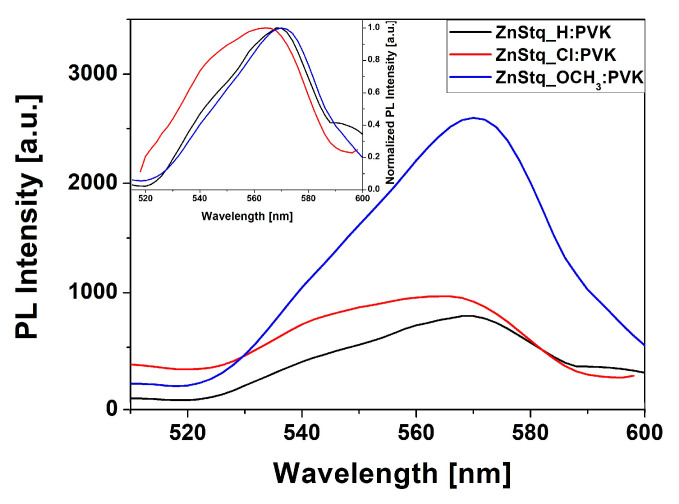
Photoluminescence spectra of ZnStq_R dispersed in PVK matrices. Insert: normalized PL spectra of ZnStq_R dispersed in PVK matrices.

**Figure 4:**
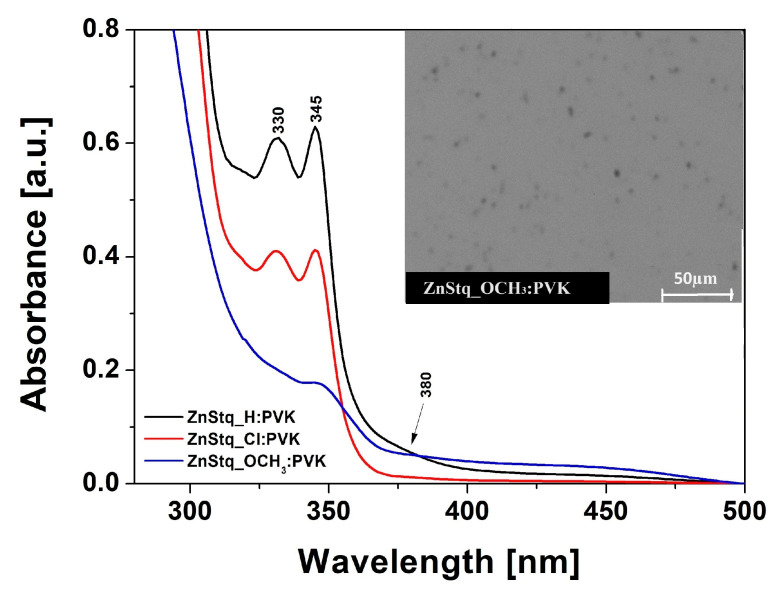
Absorption spectra of ZnStq_R:PVK thin films deposited on glass substrates. Inset: SEM image of ZnStq_OCH_3_:PVK thin film at a magnification of 200×.

The photoluminescence spectra of the ZnStq_R:PVK thin films on silicon substrates are shown in Figure 3. Thin-film measurements on Si are essential because they allow for independent measurements of the rest of the diode structure. The use of silicon reduces the contribution of reflection from the back layer of the structure, compared to aluminum, which acts as a reflector in the diode; thus, it facilitates the luminescence measurement. From Figure 4, we can observe one broad band with a maximum at 569 nm for the ZnStq_H. For the electron-withdrawing substituent (ZnStq_Cl), we observe a blue shift of the PL peak to 565 nm, and for the electron-donating substituent (ZnStq_OCH_3_), we observe a red shift to 571 nm. Barberis et al., in their work [35], described similar structures with a styryl fragment and obtained the maximum luminescence at 564 nm. From Figure 3, we can additionally note that, in the case of the sample with the methoxy group, the intensity increased twice compared to those of the ZnStq_H and ZnStq_Cl samples.

It is well known that it is possible to decrease or increase the value of the bandgap as much as possible by adjusting the HOMO (highest occupied molecular orbital)—LUMO (lowest unoccupied molecular orbital) levels by substituting appropriate donor and acceptor groups. Here, the chlorine substituent is electron-withdrawing, while –OCH_3_ is an electron-donating group. If we take into account the relative electron-withdrawing strengths of the different acceptor units, we can suppose that the –Cl strengthens the charge separation within the ZnStq_Cl molecule, when compared to the ZnStq_H molecule, thereby increasing the value of the bandgap of the ZnStq_Cl when compared to the ZnStq_H, which can be observed in Figure 4 (the blue shift of the emission in the case of the ZnStq_Cl). On the contrary, the electron-donating group –OCH_3_ reduces the charge separation, increasing the intramolecular charge transfer and the narrowing of the bandgap, which is also observed in Figure 3.

Figure 4 shows the absorption spectra of the ZnStq_R:PVK thin films deposited on glass substrates, in which bands at 315–321 nm attributed to π–π* [36], at 330 nm and 345–348 nm attributed to the π–π* bonding of the 8-hydroxyquinoline unit and at 380 nm for the ZnStq_R corresponding to the π–π* transition of the styrylquinoline unit can be observed [22]. Two bands at 330 and 345 nm are visible for the ZnStq_H and ZnStq_Cl. In the case of the ZnStq_OCH_3_, the band disappears at 330 nm, and the second band shifts to 348 nm.

The inset of Figure 4 shows an example of an SEM image of the ZnStq_OCH_3_:PVK thin film. All obtained scans show the smoothness of the surface and the even dispersion of the ZnStq_R in the PVK polymer matrix, which is very important in the case of organic light-emitting diode structures.

**Figure 5:**
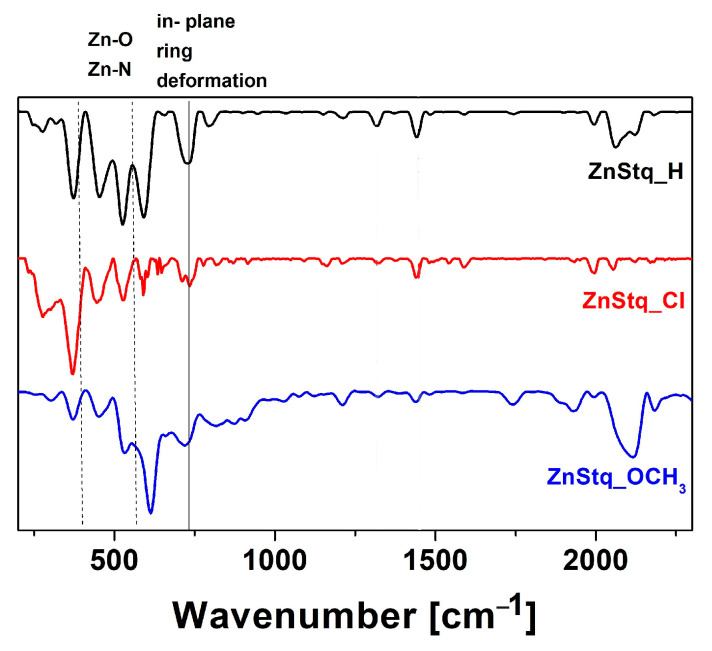
FTIR spectra for thin films of ZnStq_R in polymer matrices.

FTIR spectra for the thin films of the tested materials in the range of 200–2500 cm^−1^ are presented in Figure 5. The peaks at 400–600 cm^−1^ were associated, among others, with the Zn–O stretching vibration and Zn–N stretching vibration [36]. For the measured samples, the vibrations at 1316–1323 cm^−1^ were attributed to the quinolone group. A vibration of C–O at around 1250 cm^−1^ was also observed. Moreover the peaks at about 589–610 cm^−1^ and 730 cm^−1^ were associated with in-plane quinoline rings deformations [36]. In addition, for all the ZnStq_R, we can notice additional bands at 1650 and 1596 cm^−1^ (the C= C and C= N stretching of pyridine ring).

**Figure 6:**
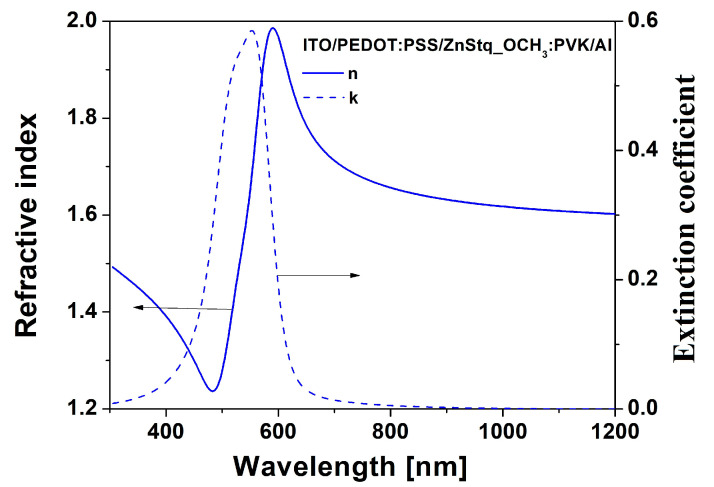
Dependence of refractive index (*n*) and extinction coefficient (*k*) on wavelength for ZnStq_OCH_3_:PVK layer in the ITO/PEDOT:PSS/ZnStq_OCH_3_:PVK/Al structure.

Examples of the dispersion features of the refractive index (*n*) and the extinction coefficient (*k*) determined from the best fits of the Tauc–Lorentz model with Gaussian oscillators to the experimental data obtained from ellipsometric measurements for the ZnStq_OCH_3_:PVK layer in the studied OLED structure are shown in Figure 6.

One can see that for *λ* > 600 nm, the *k* ≅ 0 and refractive index (*n*) show normal dispersion. At about 600 nm, the refractive index reaches its highest value at around 1.98, which decreases to 1.6 as the wavelength decreases. The maximum peak value of the extinction coefficient (*k*) is reached at about 550 nm, where the refractive index exhibits anomalous dispersion.

## 3. Materials and Methods

Poly N-vinylcarbazole (PVK, M_w_~1,000,000 g/mol) powder was purchased from Sigma-Aldrich (St. Louis, MO, USA). In turn, the powders of bis(8-hydroxyquinoline) zinc with a styryl group and different substituents were synthesized according to the procedure presented below.

### 3.1. Synthesis

In this work, two substituents with similar strengths but opposite signs on the Hammett scale were selected. The Hammett sigma constant of an electron-donating substituent such as OCH_3_ is −0.27, while the electron-withdrawing value of Cl is 0.28.

**Figure 7:**
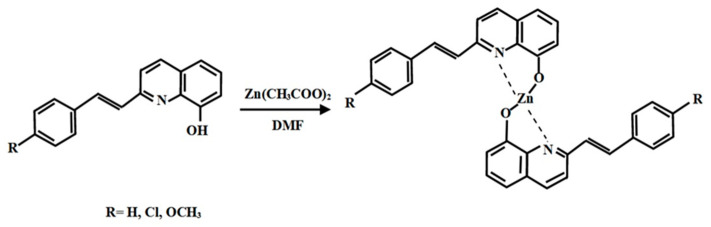
Synthetic route and chemical structure of ZnStq_R complexes, where R = H, Cl, OCH_3_.

The ligands of Stq_H, Stq_OCH_3_ and Stq_Cl were synthesized via the reaction of condensation-corresponding benzaldehydes and 2-methyl-8-quinolinol. The experimental procedure has been described elsewhere [37]. The Zn-containing complexes were prepared via reaction with corresponding Stq compounds and Zn(CH_3_COO)_2_ in DMF solution with a mole ratio of 2:1. The synthetic route and chemical structure for ZnStq complexes are shown in Figure 7.

*Zincum (II) 2-(2-phenylethenyl)quinolin-8-ol) (ZnStq_H):*

A solution of Zn(II) acetate (0.1 g, 0.54 mmol) in water (6 mL) was added to a solution of 2-(2-phenylethenyl)quinolin-8-ol) (0.28 g, 1.08 mmol) in DMF (40 mL). The reaction mixture was heated at ~100 °C and stirred 3 h under N_2_, and the complex was synthesized during the heating period. After cooling, the reaction mixture was poured into an ice-water mixture and the precipitate was filtered off, washed with water and dried. A yellow solid was obtained (yield: 50%), and the melting point was 248 °C.

^1^H NMR (400 Hz, DMSO-d 6), δ, ppm: 7.03 (d, 1H, Het), 7.27 (m, 1H, Het), 7.30–7.34 (m, 2H, Ar-H), 7.38 (m, 1H, Ar-H), 7.41 (m, 1H, =CH–), 7.42 (m, 1H, Het), 7.67–7.70 (m, 2H, Ar-H), 7.72 (m, 1H, Het), 8.07 (d, 1H,=CH–), 8.20 (d, 1H, Het). ^13^C NMR(DMSO-d6) δ: 111.7, 117.8, 121.3, 127.4, 128.0, 128.5, 128.9, 129.2, 134.6, 136.8, 138.5, 153.3, 153.7. Mass spectrum, m/z (I rel, %): 558 (18) [M]+, 556 (20), 248 (10), 247 (65), 246 (100), 216 (18), 154 (20) 144 (17). Elemental analysis (%): calcul for (C_34_H_24_N_2_O_2_Zn): C, 73.19; H, 4.34; N, 5.02; O, 5.73; Zn, 11.72; found C, 73.2; H, 4.3; N, 5.0.

*Zincum (II) 2-[2-(4-methoxyphenyl)ethenyl]quinolin-8-ol (ZnStq_OCH_3_):*

The same procedure as for ZnStq_H was used. A yellow solid was obtained (yield: 67%; M.p.: 250 °C).

^1^H NMR (400 Hz, DMSO-d6), δ, ppm: 3.83 (s, 3H, –OCH_3_), 6.95 (d, 2H, Ar-H), 7.62 (d, 2H, Ar-H), 7.26 (d, 1H, –CH=), 8.00 (d, 1H, –CH=), 7.03 (d, 1H, Het), 7.22 (m, 1H, Het), 7.34 (t, 1H, Het), 7.67 (d, 1H, Het), 8.16 (d, 1H, Het). ^13^C NMR(DMSO-d6) δ: 55.61, 111.6, 114.7, 117.9, 121.2, 126.1, 127.2, 127.9, 129.0, 129.5, 134.5, 136.8, 138.6, 153.3, 154.2, 160.17. Mass spectrum, m/z (I rel, %): 618 (10) [M]+, 278 (15), 277 (100), 276 (80), 271 (10), 262 (10), 234 (10), 233 (20), 107 (10). Elemental analysis (%): calcul for (C_36_H_28_N_2_O_4_Zn): C, 69.97; H, 4.57; N, 4.53; O, 10.36; Zn, 10.58; found C, 69.8; H, 4.4; N, 4.4.

*Zincum (II) 2-[2-(4-chlorophenyl)ethenyl]quinolin-8-ol (ZnStq_Cl):*

The same procedure as for ZnStq_H was used. A yellow solid was obtained (yield: 58%; M.p.: 289 °C).

^1^H NMR (400 Hz, DMSO-d6), δ, ppm: 7.43 (m, 2H, Ar-H), 7.70 (m, 2H, Ar-H), 7.33 (m, 1H, –CH=), 8.08 (d, 1H, –CH=), 7.05 (d, 1H, Het), 7.27 (m, 1H, Het), 7.35 (m, 1H, Het), 7.71 (m, 1H, Het), 8.20 (d, 1H, Het). ^13^C NMR(DMSO-d6) δ: 111.6, 118.0, 121.4, 127.6, 128.2, 129.2, 129.3, 133.3, 135.8, 137.0, 138.6, 153.4, 153.5. Mass spectrum, m/z (I rel, %): 626 (15) [M]+, 281 (100), 170 (5), 144 (5), 113 (20), 74 (15), 50(20). Elemental analysis (%): calcul for (C_34_H_22_Cl_2_N_2_O_2_Zn): C, 65.14; H, 3.54; Cl, 11.31; N, 4.47; O, 5.10; Zn, 10.43; found C, 65.1; H, 3.5; N, 4.4.

### 3.2. OLED Preparation

The glass substrates with an indium tin oxide (ITO) layer (*L_ITO_* = 118 nm, *R_s_* = 15 W/sq.) were purchased from Sigma Aldrich and cleaned in an ultrasonic bath using isopropanol (P 99.5%, Sigma-Aldrich), acetone (ACS reagent, P 99.5%, Sigma-Aldrich), tetrahydrofuran (THF, anhydrous, 99.9% Sigma-Aldrich), detergent and distilled water. The ITO layer was used as an optically transparent anode. An aqueous solution of PEDOT:PSS (1.1 wt% of poly(3,4-ethylenedioxythiophene):polystyrene sulfonic acid dispersed in H_2_O), as a *p*-type organic semiconductor and hole transport material, was deposited on the ITO/glass via the spin-coating method (*L_PEDOT:PSS_* ~ 60 nm). The obtained PEDOT:PSS layer (surfactant-free, high-conductive grade) was dried under vacuum at 100 °C for 30 min. Then, the solution of ZnStq_R:PVK dissolved in THF was deposited on the PEDOT:PSS films under an argon atmosphere via the spin-coating technique (*L_ZnStq_R_*~100 nm). The obtained structure was then dried at 70 °C for 30 min to remove traces of THF. Finally, a 100 nm thick aluminum (Al) layer was deposited on the ITO/PEDOT:PSS/ZnStq_R:PVK structure via vacuum vapor deposition under medium vacuum conditions (10^−6^ bar) [38]. The obtained OLED (ITO/PEDOT:PSS/ZnStq_R:PVK/Al) with an active ZnStq_R:PVK layer of about 24 mm^2^ was fabricated as presented in [39].

### 3.3. Thin-Film Preparation for Optical Characterization

To prepare the ZnStq_R:PVK thin films, we first prepared a 5 wt% solution of PVK in dichloroethane (HPLC ≥ 99.8%, Sigma Aldrich). In the next stage, 0.02 g of the corresponding ZnStq_R powder, synthesized as described in Section 3.1, was added to 10 ml of the prepared solution. To ensure the thorough mixing of the powder with the polymer, the mixture was placed in an ultrasonic bath for approximately 30 min. To obtain thin layers, the ZnStq_R:PVK mixture was deposited on silicon and glass substrates using the spin-coating method (Spin-coater Laurell WS-650SZ-6NPP/A1/AR1/OND, 1600 rpm). The layers were then dried at room temperature in an ambient atmosphere for 24 h.

### 3.4. Experimental Methods

In our work, the electroluminescence of the obtained organic light-emitting diode structures was measured at forward-bias voltages (i.e., ITO, positive; Al, negative) in the range of 9–15 V. The emitted light was collected and analyzed via a CCD spectrophotometer MS125 (Oriel Instruments Corp., Andover, MA, USA) with a spectral resolution of 2 nm in the range of 400–850 nm. The fabricated device was exposed to air without encapsulation. For the photo-physical characterization of the thin film, the following measurements were performed: FTIR, UV-VIS spectroscopy, photoluminescence and ellipsometric spectroscopy.

FTIR spectra of the ZnStq_R:PVK thin films were measured using FT-IR Vertex 70 V with a Hyperion 1000/2000 microscope by Bruker Optik from 200 to 2500 cm^−1^. UV-VIS measurements of the ZnStq_R:PVK thin films deposited on glass substrates were made at room temperature using a Specord 200 spectrometer in the range of 270–500 nm. The photoluminescent (PL) signals of the studied thin films were registered via a Jasco spectrofluorometer (model FP-8200,Tokyo, Japan) with an additional solid-state adapter in the range of 500–600 nm (*λ_exc_*_._ = 320 nm, Xe lamp). Optical parameters, such as the extinction coefficient (*k*) and refractive index (*n*), of the ZnStq_R:PVK thin films were determined using the spectroscopic ellipsometer Woollam M2000 (J.A. Woollam Co., Inc., Lincoln, NE, USA) and CompleteEASE 5.15 software. Ellipsometric angles (Δ and Ψ), defined from the ratio of the amplitude reflection coefficients for p and s polarizations, were measured for three angles (i.e., 60°, 65° and 70°) of the light incidence in the range from 300 nm to 1200 nm. The sought parameters of the ZnStq_R:PVK layers were determined using the Tauc–Lorentz model with Gaussian oscillators, which was fitted to the experimental data [34]. In addition, the ZnStq_R:PVK layers in the ITO/PEDOT:PSS/ZnStq_R:PVK structure were measured via ellipsometric spectroscopy to determine the thickness of the emission layers in these OLEDs. All measurements were carried out at room temperature. 

## 4. Conclusions

In this work, we fabricated organic light-emitting diodes (OLEDs) based on newly synthesized bis(8-hydroxyquinoline) zinc with a styryl fragment (ZnStq_R, where R= H, Cl, OCH_3_) in poly(9-vinylcarbazole) matrix (PVK). The obtained OLEDs exhibited strong yellow electroluminescent emissions at 590, 587 and 578 nm for the ZnStq_H:PVK, ZnStq_Cl:PVK and ZnStq_OCH_3_:PVK, respectively. We found that our OLEDs exhibited the highest maximal values of the brightness: approximately 2595 cd/m^2^ with a current efficiency of 1.21 cd/A for the ZnStq_H, 1793 cd/m^2^ with a current efficiency of 0.85 cd/A for the ZnStq_Cl and 2244 cd/m^2^ with a current efficiency of 1.24 cd/A for the ZnStq_OCH_3_. After that, we prepared thin films of all the synthetized ZnStq_R samples on glass and silicon substrates using the spin-coating method. In this case, the maximum photoluminescence value was observed at 569 nm for the ZnStq_H, while for the acceptor substituent (ZnStq_Cl), we observed a blue shift to 565 nm. In contrast, the donor substituent (ZnStq_OCH_3_) caused a red shift to 571 nm. The absorption spectra show bands at 318 nm, attributed to π–π*, bands at 330 and 345 nm, attributed to the π–π* bonding of the 8-hydroxyquinoline unit and bands at 370 nm for the ZnStq_R, corresponding to the π–π* transition of the styrylquinoline unit. The composition of the studied thin layers containing both components was confirmed via the FTIR method.

As a result, the OLED with the ZnStq_OCH_3_:PVK film as the active layer showed narrow EL spectra with an *FWHM* of 59 nm, maximum brightness of 2244 cd/m^2^ and maximum current efficiency of 1.24 cd/A, with a turn-on voltage of 6.94 V and threshold voltage of 7.35 V; thus, it is a promising device for commercial organic light-emitting diode application.

## Figures and Tables

**Table 1:** Selected parameters of the produced OLED structure devices: *λ_ELmax_*—peak position of maximum EL emission; *U_on_*—turn-on voltage; *U_T_*—threshold voltage; *B_max_*—maximal brightness; *Max CE*—maximum current efficiency; *FWHM*—full width at half maximum; *d*—thickness of an emissive layer.

Structure: ITO/PEDOT:PSS/	*λ_ELmax_* (nm)	*U_on_* (V)	*U_T_* (V)	*B_max_* (cd/m^2^)	*Max CE* (cd/A)	*FWHM* (nm)	*d* (nm)
/ZnStq_H:PVK/Al	590 ± 5	5.38 ± 0.01	6.35 ± 0.01	2595 ± 10	1.21 ± 0.01	82 ± 2	106 ± 1.2
/ZnStq_Cl:PVK/Al	587 ± 5	5.67 ± 0.01	7.80 ± 0.01	1793 ± 10	0.85 ± 0.01	59 ± 2	98 ± 1.1
/ZnStq_OCH_3_:PVK/Al	578 ± 5	6.94 ± 0.01	7.35 ± 0.01	2244 ± 10	1.24 ± 0.01	59 ± 2	101 ± 1.3

## Data Availability

Data supporting the results of this study are available upon request only.
